# Ethnic differences in the risk of caesarean section: a Danish population-based register study 2004–2015

**DOI:** 10.1186/s12884-019-2331-6

**Published:** 2019-06-04

**Authors:** Trine Damsted Rasmussen, Sarah Fredsted Villadsen, Per Kragh Andersen, Tine Dalsgaard Clausen, Anne-Marie Nybo Andersen

**Affiliations:** 1 Section of Epidemiology, Department of Public Health, University of Copenhagen, Øster Farimagsgade 5, Postbox 2099, 1014 Copenhagen K, Denmark; 2 Section of Social Medicine, Department of Public Health, University of Copenhagen, Øster Farimagsgade 5, Postbox 2099, 1014 Copenhagen K, Denmark; 3 Section of Biostatistics, Department of Public Health, University of Copenhagen, Øster Farimagsgade 5 opg. B, Postbox 2099, 1014 Copenhagen K, Denmark; 4 Department of Gynecology and Obstetrics, Nordsjællands Hospital, Dyrehavevej 29, 3400 Hillerød, Denmark

**Keywords:** Caesarean section, Immigrants, Ethnicity, Disparities, Risk factors

## Abstract

**Background:**

Studies have shown differences in the risk of caesarean section (CS) between ethnic minority groups. This could be a marker of unequal health care. The aim of this study was to investigate differences in the risk of CS between immigrants of various origins in Denmark, where all health care is free and easy to access, and Danish-born women. A further aim was to determine the possible influence of known risk factors for CS.

**Methods:**

The design was a population-based register study using national Danish registers and included all live- and stillborn singleton deliveries by primiparous women in Denmark from 2004 to 2015. The total study population consisted of 298,086 births, including 25,198 births to women from the 19 largest immigrant groups in Denmark.

Multinomial logistic regression analysis was used to estimate relative risk ratios (RRR) of emergency and planned CS, using vaginal delivery (VD) as reference, in immigrant women compared to Danish-born women. A number of known risk factors were included separately.

**Results:**

Women from Turkey, the Philippines, Thailand, Somalia, Vietnam, Iran and Afghanistan had a statistically significant elevated risk ratio of emergency CS vs. VD compared to Danish-born women; adjusted RRR’s ranging 1.15–2.19. The risk ratio of planned CS vs. VD was lower among the majority of immigrant groups, however higher among women from Poland, Thailand and Iran, when compared to Danish-born women. None of the studied explanatory variables affected the risk ratio of planned CS vs. VD, whereas maternal height contributed with varying strength to the risk ratio of emergency CS vs. VD for all immigrant groups.

**Conclusion:**

Substantial variations in CS risks by maternal country of birth were documented. Some of the disparities in emergency CS seem to be explained by maternal height.

**Electronic supplementary material:**

The online version of this article (10.1186/s12884-019-2331-6) contains supplementary material, which is available to authorized users.

## Background

The use of medical interventions in childbirth, including caesarean section (CS), has increased during the past decades [1]. In Brazil and Iran CS rates have reached levels of around 50% of all deliveries [2]. In Denmark the CS rate increased from 13% in 1995 [3] to 21% in 2015 [2]. Even though a CS can be a lifesaving procedure when medically indicated, the World Health Organization (WHO) has assessed that national CS rates exceeding 10–15% are not associated with reduced maternal and perinatal mortality [4]. Although today a CS is considered a safe procedure in most countries, it is still associated with an increased number of short and long-term complications for both the mother and the child [4]. Potential pathways leading to CS are likely multifactorial and interrelated [5] and combine biological, medical, social and cultural conditions as well as quality of care issues [5, 6].

International migration has grown rapidly during the past 15 years. Globally, women represent around one half of all migrants [7] and in many Western European countries, around one-fifth of all births are to immigrant women [8]. Likewise, the number of immigrants in Denmark has increased in recent decades; the number of non-Western immigrants increased more than 5 times from 1986 to 2016 [9].

Several international studies have shown differences in the number of births delivered by CS between migrants and national born women in Western industrialized countries [10]. A systematic review and meta-analysis from 2013 and a review from 2015 revealed consistently higher overall CS rates among immigrant women from Sub-Saharan Africa, Somalia and South Asia compared to non-migrants and additionally higher rates of emergency CS among women from North Africa/ Middle East and Latin America [6, 10].

Ethnic disparities in CS could be a marker of unequal health care and could indicate both under- and overuse, which is why both high and low rates of CS might be of concern. Underuse of planned and overuse of emergency CS in the same group of women could indicate issues related to suboptimal communication and antenatal care.

Thus, the overall aim of this study was to investigate differences in the risk of CS in Denmark between the largest immigrant groups and Danish-born women in the period 2004–2015. Additionally, the aim was to investigate whether known risk factors for CS could contribute to and explain potential differences.

## Methods

### Design and study population

We conducted a population-based register study including all live- and stillborn singleton births with gestational age ≥ 22 weeks delivered by Danish-born women and women from the 19 largest immigrant groups in Denmark in the period 2004–2015. Danish-born women were included as the reference group. We chose only to include primiparous women to avoid the influence of delivery mode and outcome of previous pregnancies.

Data was drawn from the Danish Medical Birth Registry and linked to socioeconomic and demographic data including information about maternal country of origin and immigrant status from national registries at Statistics Denmark, using unique personal identification numbers.

Women with an unspecified type of CS that could not be classified as either emergency or planned were excluded (*n* = 50). After this exclusion, the total study population for analysis consisted of 298,086 births, including 25,198 births by immigrant mothers.

### Main outcome measure

The outcome measure was delivery by CS classified as either emergency or planned with vaginal delivery (VD) as reference. Information about type of CS was obtained from the Danish Medical Birth Registry, based on International Classification of Disease codes (ICD-10) and operation codes. Emergency CS was based on the following codes: ICD-10 O821 and operation codes KMCA10 (A, D, E) and KMCA12 (A, B). Planned CS was based on ICD-10 O820 and operation codes KMCA10B and KMCA11.

### Exposure

The main exposure was maternal country of birth. According to Statistics Denmark, immigrants are defined as being born abroad and having no parents who are both: (1) born in Denmark and (2) Danish citizens [9]. Descendants are defined by the same criteria except they are born in Denmark. Danish-born women were used as the reference group and included descendants. Deliveries by descendants accounted for 2% of the births by Danish-born women.

For a small percentage of women, new information about parental origin or citizenship resulted in change in immigrant status and country of origin. For these women we used the latest documented information.

### Other explanatory variables

We a priori selected a number of explanatory variables known to be associated with maternal country of birth and the risk of CS including year of birth, maternal age, gestational age, pre-pregnancy BMI, maternal height, fetal birth weight and medical conditions such as gestational hypertensive disorders (preeclampsia or eclampsia) and diabetes. Information about these variables was drawn from the Danish Medical Birth Registry.

Year of birth was the only variable considered as a potential confounder as both the composition of the various immigrant groups and the annual number of CS, possibly caused by changes in clinical guidelines, changed over the years. All other covariates were considered as explanatory factors.

Maternal age at delivery was divided into three categories: ≤ 24, 25–34 and ≥ 35 years and gestational age as preterm (week 22 + 0–36 + 6), term (week 37 + 0–41 + 6) and postterm (≥ 42 + 0). Prepregnancy BMI was divided into the following four categories: underweight (< 18.5), normal weight (18.5–24.9), overweight (25–29.9) and obese (≥ 30). Maternal height was used as a continuous variable in cm. 3–12% of women from all countries of origin had missing information about maternal pre-pregnancy BMI and height.

Information about diabetes and gestational hypertensive disorders was coded as yes/no. Information about diabetes included both type 1, type 2 and gestational diabetes and was based on ICD-10 code DO24 and additional sub codes. The variable gestational hypertensive disorders was based on ICD-10 codes O13-O15 (hypertension, preeclampsia and eclampsia). Fetal birth weight was divided into the categories: < 2500 g., 2500–4500 g. and > 4500 g. Fetal birth weight was also listed as a mean value.

Maternal level of highest educational attainment was categorized as < 10, 10–12, > 12 years and no information. The last category was formed due to a high number of missing values among immigrant women. Paternal origin was divided into the categories Danish-born and immigrant/ descendant.

### Statistical analysis

Descriptive analyses were conducted to examine the distribution of explanatory variables according to maternal country of birth. Multinomial logistic regression analysis was used to estimate ratios between relative risks (RRR) of respectively emergency and planned CS vs. VD, with corresponding 95% confidence intervals (CI), for immigrant women and Danish-born women. All analyses were complete case analyses and adjusted for year of birth. The regression model adjusted for year of birth was additionally adjusted separately for maternal age, length of gestation, diabetes, gestational hypertensive disorders, fetal birth weight, maternal prepregnancy BMI and maternal height, to examine to what extent these factors could explain ethnic differences in risk of CS.

All analyses were conducted using STATA version 14.1.

## Results

Ex-Yugoslavian and Polish women accounted for the largest proportion of births to immigrants, with 2699 and 2685 deliveries respectively (Tables 1 and 2).

**Table 1:** Number of deliveries by primiparous women and explanatory factors by maternal country of birth: Denmark 2004–2015

Total number of deliveries by maternal country of birth	Denmark *n* = 272,888	Ex. Yugoslavia *n* = 2699	Poland *n* = 2685	Turkey *n* = 1856	Iraq *n* = 1663	Germany *n* = 1500	Norway *n* = 1486	Sweden *n* = 1403	Romania *n* = 1346	China *n* = 1292
Maternal age at delivery (years)										
≤ 24	19.9	33.3	19.2	33.2	51.9	12.0	10.4	8.5	17.1	10.1
25–34	69.4	59.5	72.1	59.1	42.5	67.6	76.6	73.0	74.1	76.4
≥ 35	10.7	7.2	8.7	7.7	5.7	20.4	13.1	18.5	8.8	13.5
Gestational age at delivery (weeks)										
Preterm (22 + 0–36 + 6)	6.6	5.9	4.8	5.9	5.8	5.6	6.3	5.1	5.1	4.5
Term (37 + 0–41 + 6)	87.5	88.7	89.8	88.5	90.0	89.2	87.3	88.5	92.1	91.5
Postterm (≥ 42 + 0)	5.8	5.4	5.3	5.4	4.2	5.0	6.4	6.3	2.7	4.0
Missing	0.1	0.1	0.1	0.2	0.1	0.2	0.0	0.1	0.1	0.0
BMI										
Underweight < 18.5	4.2	5.7	7.0	3.2	6.0	3.7	4.9	5.5	9.6	16.4
Normalweight 18.5–24.9	62.3	70.3	70.8	58.6	61.9	69.5	74.4	76.6	69.0	70.5
Overweight 25–29.9	19.3	13.9	14.3	20.1	19.1	16.7	14.3	11.4	14.7	7.1
Obese ≥30	11.0	5.7	4.3	6.7	7.9	7.1	4.2	3.1	4.4	1.3
Missing	3.3	4.4	3.6	11.4	5.1	3.1	2.3	3.5	2.3	4.6
Maternal height (mean)	168.5	166.1	166.8	163.3	161.7	168.7	168.8	168.3	164.7	161.9
Missing	2.7	3.7	3.1	10.6	4.3	2.9	2.2	2.7	2.2	3.7
Diabetes										
No	97.2	96.9	97.4	94.2	95.2	97.5	98.0	98.9	96.7	93.9
Yes	2.8	3.1	2.6	5.8	4.8	2.5	2.0	1.1	3.3	6.1
Gestational hypertensive disorders										
No	93.7	96.4	95.2	96.3	97.1	95.5	95.5	93.7	96.9	98.2
Yes	6.3	3.6	4.8	3.7	2.9	4.5	4.5	6.3	3.1	1.8
Birthweight										
< 2500 g.	4.5	4.4	3.3	5.0	4.9	4.2	4.2	3.9	4.2	2.5
2500–4500 g.	92.6	92.5	93.8	90.5	92.5	93.4	93.4	92.5	93.3	94.4
> 4500 g.	1.9	1.7	1.6	2.0	0.8	1.5	1.4	2.2	1.2	2.2
Missing	1.0	1.4	1.3	2.6	1.8	0.9	1.1	1.4	1.3	0.9
Mean (g.)	3432.9	3405.7	3415.2	3362.7	3285.2	3406.7	3451.1	3444.9	3367.2	3443.6
Maternal education (years)										
< 10	16.0	24.4	5.3	34.3	46.4	5.4	3.6	4.2	2.8	7.0
10–12	38.4	34.5	13.5	26.0	24.4	17.2	15.1	16.2	10.1	13.6
> 12	44.5	22.1	18.9	12.3	10.9	35.4	47.9	41.7	18.9	34.1
No information	1.1	19.0	62.4	27.4	18.2	42.0	33.4	37.9	68.2	45.3
Paternal origin										
Danish	90.7	14.0	30.1	4.5	4.2	59.2	78.1	76.0	23.3	35.3
Immigrant/ descendant	6.0	77.0	65.3	87.5	82.1	36.8	19.6	20.6	72.6	60.8
Missing	3.4	9.0	4.7	8.0	13.8	4.0	2.3	3.4	4.1	3.9

Frequencies are given in percentages and in rounded estimates

**Table 2:** Number of deliveries by primiparous women and explanatory factors by maternal country of birth: Denmark 2004–2015

Total number of deliveries by maternal country of birth	Denmark *n* = 272,888	Philippines *n* = 1127	Thailand *n* = 1088	Pakistan *n* = 1070	Somalia *n* = 956	Vietnam *n* = 953	Lebanon *n* = 926	Iceland *n* = 910	Iran *n* = 906	Afghanistan *n* = 860	Morocco *n* = 472
Maternal age at delivery (years)											
≤ 24	19.9	10.1	15.3	27.7	48.2	11.8	52.5	24.2	12.0	39.3	16.5
25–34	69.4	78.0	64.2	65.9	44.9	77.1	42.0	69.5	69.1	55.7	65.3
≥ 35	10.7	11.9	20.6	6.5	6.9	11.1	5.5	6.4	18.9	5.0	18.2
Gestational age at delivery (weeks)											
Preterm (22 + 0– 36 + 6)	6.6	7.5	7.8	7.2	6.7	7.9	5.8	5.6	5.3	5.1	4.5
Term (37 + 0–41 + 6)	87.5	88.6	89.9	89.4	80.5	89.6	90.1	88.7	91.1	91.1	86.8
Postterm (≥ 42 + 0)	5.8	3.8	2.3	3.5	12.7	2.3	4.1	5.7	3.5	3.6	8.5
Missing	0.1	0.0	0.0	0.0	0.1	0.2	0.0	0.0	0.1	0.2	0.2
BMI											
Underweight < 18.5	4.2	12.6	14.6	10.7	12.0	16.0	6.4	3.9	5.5	8.3	4.2
Normalweight 18.5–24.9	62.3	67.7	68.9	54.4	53.9	74.1	63.4	61.8	66.7	65.2	56.4
Overweight 25–29.9	19.3	11.9	8.7	14.8	19.0	4.8	17.1	20.8	17.0	15.5	22.7
Obese ≥30	11.0	2.8	2.1	8.1	7.6	1.1	6.8	10.2	6.3	3.0	6.8
Missing	3.3	5.0	5.6	12.1	7.4	4.1	6.4	3.4	4.5	8.0	10.0
Maternal height (mean)	168.5	155.4	158.4	160.7	164.7	156.9	162.5	167.8	162.9	160.3	163.5
Missing	2.7	4.4	5.1	10.4	5.7	3.2	5.6	3.0	4.0	6.6	8.5
Diabetes											
No	97.2	94.2	95.6	92.5	95.9	96.0	97.4	98.7	95.5	94.9	91.1
Yes	2.8	5.8	4.4	7.5	4.1	4.0	2.6	1.3	4.5	5.1	8.9
Gestational hypertensive disorders											
No	93.7	95.3	97.0	95.0	91.2	97.7	97.5	93.7	96.9	97.1	96.8
Yes	6.3	4.7	3.0	5.1	8.8	2.3	2.5	6.3	3.1	2.9	3.2
Birthweight											
< 2500 g.	4.5	5.8	5.9	9.2	7.4	6.5	4.1	4.0	5.0	5.0	3.6
2500–4500 g.	92.6	91.3	91.5	87.9	90.2	91.8	92.6	91.8	92.7	89.9	92.8
> 4500 g.	1.9	1.5	0.9	0.3	0.8	0.6	0.9	3.3	1.3	2.3	1.7
Missing	1.0	1.4	1.7	2.6	1.6	1.1	2.5	1.0	1.0	2.8	1.9
Mean (g.)	3432.9	3326.4	3313.5	3141.9	3212.9	3212.4	3289.3	3524.2	3344.7	3347.7	3409.8
Maternal education (years)											
< 10	16.0	9.8	27.0	17.9	52.2	25.3	41.0	11.1	16.0	32.8	19.5
10–12	38.4	18.1	22.4	16.9	25.5	25.5	27.9	17.5	25.7	27.6	24.8
> 12	44.5	14.6	9.2	10.7	4.3	21.8	11.3	31.5	34.0	8.7	12.3
No information	1.1	57.6	41.4	54.6	18.0	27.4	19.8	39.9	24.3	30.9	43.4
Paternal origin											
Danish	90.7	79.5	82.0	2.1	4.5	28.3	5.5	37.1	26.1	2.7	10.0
Immigrant/ descendant	6.0	16.9	14.9	90.8	70.0	66.5	78.1	58.5	70.5	85.8	85.4
Missing	3.4	3.6	3.1	7.2	25.5	5.1	16.4	4.4	3.4	11.5	4.7

Frequencies are given in percentages and in rounded estimates

Maternal age at delivery varied among ethnic groups. Around 50% of women from Iraq, Somalia and Lebanon were 24 years or younger when having their first child, whereas women from Germany, Thailand and Iran had the highest number of first-time deliveries above the age of 35.

Compared to all other groups, women from Somalia had the highest frequency of postterm deliveries.

Being underweight (BMI < 18.5) was most prevalent among women from China, Vietnam, Thailand and the Philippines, while being obese (BMI ≥ 30) was most prevalent among Icelandic and Danish-born women.

Women from Vietnam, Thailand and the Philippines had the lowest mean maternal height (mean height ≈ 155 cm).

Diabetes in pregnancy was most prevalent among women from Morocco and Pakistan, whereas women from Somalia, Sweden, Iceland and Danish-born women had the highest prevalence of gestational hypertensive disorders.

Except for women from Germany, Norway, Sweden, Iceland, China and Iran, educational level was lower for all other immigrant groups compared to Danish-born women. However, information about highest educational attainment was missing for a substantial number of immigrants (ranging from 18 to 68%).

The large majority of children born by non-Western immigrant mothers had fathers who were immigrants or descendants, except for children born by women from Thailand and the Philippines, where 80% of the fathers were Danish-born. For children of Somali-born women 25% had no information about paternal origin.

Table 3 shows the estimated RRRs and 95% CIs for emergency and planned CS respectively among the 19 largest groups of immigrants compared to Danish-born women, adjusted for year of birth. The risk ratio of emergency CS vs. VD was statistically significantly increased for women from Turkey (RRR: 1.15, 95% CI = 1.02–1.30), the Philippines (RRR: 2.19, 95% CI = 1.92–2.49), Thailand (RRR: 1.61, 95% CI = 1.40–1.86), Somalia (RRR: 1.74, 95% CI = 1.50–2.01), Vietnam (RRR: 1.26, 95% CI = 1.07–1.47), Iran (RRR: 1.66, 95% CI = 1.42–1.95) and Afghanistan (RRR: 1.29, 95% CI = 1.09–1.53) compared to Danish-born women.

**Table 3:** Relative risk ratios (RRR) and 95% confidence intervals for respectively emergency caesarean section (CS) and planned CS versus vaginal delivery among primiparous women by maternal country of birth: Denmark 2004–2015

Maternal country of birth	Total number of deliveries n	Emergency CS (%)^a^	Planned CS (%)^a^	Vaginal delivery (%)^a^	Emergency CS	Planned CS
					Adjusted for year of birth	Adjusted for year of birth
Denmark	272,888	16.0	5.4	78.5	1.00 (Ref.)	1.00 (Ref.)
Ex-Yugoslavia	2699	15.9	5.1	79.1	0.98 (0.89–1.09)	0.93 (0.78–1.11)
Poland	2685	15.0	6.6	78.4	0.95 (0.85–1.05)	1.22 (1.05–1.43)
Turkey	1856	18.4	3.6	78.0	1.15 (1.02–1.30)	0.66 (0.52–0.85)
Iraq	1663	16.5	4.0	79.5	1.02 (0.89–1.16)	0.72 (0.57–0.93)
Germany	1500	15.1	5.5	79.5	0.93 (0.81–1.08)	1.00 (0.80–1.25)
Norway	1486	12.3	4.8	82.9	0.73 (0.62–0.85)	0.83 (0.66–1.06)
Sweden	1403	14.8	6.4	78.8	0.92 (0.79–1.07)	1.18 (0.95–1.46)
Romania	1346	15.8	6.3	77.9	1.01 (0.87–1.17)	1.18 (0.95–1.48)
China	1292	15.7	3.7	80.6	0.96 (0.83–1.12)	0.67 (0.50–0.90)
Philippines	1127	29.0	5.3	65.7	2.19 (1.92–2.49)	1.18 (0.91–1.53)
Thailand	1088	23.1	6.8	70.1	1.61 (1.40–1.86)	1.40 (1.11–1.78)
Pakistan	1070	17.0	3.8	79.2	1.06 (0.90–1.24)	0.70 (0.51–0.96)
Somalia	956	25.4	2.9	71.7	1.74 (1.50–2.01)	0.59 (0.41–0.86)
Vietnam	953	19.7	3.5	76.8	1.26 (1.07–1.47)	0.65 (0.46–0.92)
Lebanon	926	12.5	2.8	84.7	0.72 (0.59–0.88)	0.48 (0.32–0.71)
Iceland	910	15.3	2.5	82.2	0.91 (0.76–1.09)	0.44 (0.29–0.67)
Iran	906	22.7	9.9	67.3	1.66 (1.42–1.95)	2.14 (1.71–2.67)
Afghanistan	860	20.0	3.6	76.4	1.29 (1.09–1.53)	0.68 (0.48–0.98)
Morocco	472	18.6	3.6	77.8	1.17 (0.93–1.48)	0.67 (0.41–1.09)

^a^Stated as a percentage of the total number of deliveries

The majority of immigrant women had a significant lower risk ratio of planned CS vs. VD compared to Danish-born women, with adjusted RRR’s ranging from 0.44 to 0.72. Women from Poland (RRR: 1.22, 95% CI = 1.05–1.43), Thailand (RRR: 1.40, 95% CI = 1.11–1.78) and Iran (RRR: 2.14, 95% CI = 1.71–2.67) were the only groups with a statistically significantly increased risk ratio of planned CS vs. VD relative to Danish-born women.

Table 4 shows that individual adjustments for maternal age, gestational age, diabetes, gestational hypertensive disorders and fetal birth weight did not considerably change the estimates for emergency CS.

**Table 4:** Relative risk ratios (RRR) and 95% confidence interval for emergency caesarean section (CS) versus vaginal delivery among primiparous women by maternal country of birth: Denmark 2004–2015

Maternal country of birth	Total number of deliveries n	Emergency CS (%)^a^	Vaginal delivery (%)^a^	Adjusted for year of birth^b^	Adjusted for maternal age^b^	Adjusted for gestational age^b^	Adjusted for diabetes^b^	Adjusted for GHD^bc^	Adjusted for birthweight^b^	Adjusted for BMI^b^	Adjusted for height^b^
Denmark	272,888	16.0	78.5	1.00 (Ref.)	1.00 (Ref.)	1.00 (Ref.)	1.00 (Ref.)	1.00 (Ref.)	1.00 (Ref.)	1.00 (Ref.)	1.00 (Ref.)
Ex-Yugoslavia	2699	15.9	79.1	0.98 (0.89–1.09)	1.05 (0.94–1.16)	1.00 (0.89–1.11)	0.98 (0.89–1.09)	1.02 (0.92–1.14)	0.99 (0.89–1.10)	1.06 (0.95–1.18)	0.89 (0.80–0.99)
Poland	2685	15.0	78.4	0.95 (0.85–1.05)	0.96 (0.86–1.07)	0.96 (0.86–1.07)	0.95 (0.85–1.06)	0.97 (0.87–1.08)	0.97 (0.87–1.08)	1.04 (0.93–1.16)	0.88 (0.79–0.99)
Turkey	1856	18.4	78.0	1.15 (1.02–1.30)	1.22 (1.08–1.38)	1.17 (1.04–1.32)	1.13 (1.00–1.27)	1.20 (1.06–1.35)	1.15 (1.02–1.30)	1.19 (1.05–1.35)	0.93 (0.82–1.05)
Iraq	1663	16.5	79.5	1.02 (0.89–1.16)	1.16 (1.02–1.31)	1.05 (0.92–1.20)	1.00 (0.88–1.14)	1.07 (0.94–1.22)	1.03 (0.90–1.17)	1.06 (0.93–1.22)	0.77 (0.67–0.88)
Germany	1500	15.1	79.5	0.93 (0.81–1.08)	0.86 (0.75–1.00)	0.95 (0.82–1.10)	0.94 (0.81–1.08)	0.96 (0.83–1.11)	0.95 (0.82–1.10)	0.97 (0.84–1.12)	0.94 (0.82–1.09)
Norway	1486	12.3	82.9	0.73 (0.62–0.85)	0.70 (0.59–0.81)	0.72 (0.62–0.84)	0.73 (0.63–0.85)	0.74 (0.64–0.87)	0.74 (0.63–0.86)	0.79 (0.67–0.92)	0.74 (0.63–0.87)
Sweden	1403	14.8	78.8	0.92 (0.79–1.07)	0.85 (0.73–0.99)	0.92 (0.80–1.07)	0.93 (0.80–1.08)	0.92 (0.79–1.07)	0.90 (0.77–1.05)	1.00 (0.86–1.16)	0.90 (0.77–1.05)
Romania	1346	15.8	77.9	1.01 (0.87–1.17)	1.02 (0.88–1.18)	1.05 (0.90–1.22)	1.01 (0.87–1.17)	1.07 (0.92–1.24)	1.02 (0.88–1.19)	1.11 (0.96–1.29)	0.86 (0.74–1.00)
China	1292	15.7	80.6	0.96 (0.83–1.12)	0.92 (0.79–1.07)	1.00 (0.86–1.17)	0.94 (0.81–1.10)	1.03 (0.89–1.20)	0.99 (0.85–1.15)	1.16 (0.99–1.35)	0.73 (0.63–0.86)
Philippines	1127	29.0	65.7	2.19 (1.92–2.49)	2.13 (1.87–2.42)	2.23 (1.95–2.54)	2.15 (1.89–2.45)	2.27 (1.99–2.58)	2.24 (1.96–2.56)	2.53 (2.21–2.89)	1.25 (1.10–1.44)
Thailand	1088	23.1	70.1	1.61 (1.40–1.86)	1.51 (1.31–1.75)	1.66 (1.43–1.92)	1.60 (1.38–1.84)	1.70 (1.47–1.96)	1.63 (1.41–1.89)	1.91 (1.65–2.21)	1.06 (0.92–1.23)
Pakistan	1070	17.0	79.2	1.06 (0.90–1.24)	1.11 (0.94–1.30)	1.07 (0.91–1.26)	1.02 (0.87–1.20)	1.08 (0.92–1.27)	1.01 (0.85–1.19)	1.08 (0.91–1.29)	0.73 (0.62–0.87)
Somalia	956	25.4	71.7	1.74 (1.50–2.01)	1.95 (1.68–2.26)	1.65 (1.42–1.91)	1.72 (1.49–2.00)	1.70 (1.47–1.97)	1.72 (1.48–1.99)	1.90 (1.63–2.21)	1.50 (1.29–1.75)
Vietnam	953	19.7	76.8	1.26 (1.07–1.47)	1.22 (1.04–1.44)	1.29 (1.10–1.52)	1.25 (1.06–1.46)	1.33 (1.13–1.56)	1.24 (1.05–1.46)	1.52 (1.29–1.79)	0.78 (0.66–0.92)
Lebanon	926	12.5	84.7	0.72 (0.59–0.88)	0.82 (0.67–1.00)	0.74 (0.61–0.90)	0.72 (0.59–0.88)	0.76 (0.62–0.92)	0.74 (0.61–0.90)	0.77 (0.63–0.94)	0.56 (0.46–0.68)
Iceland	910	15.3	82.2	0.91 (0.76–1.09)	0.94 (0.78–1.13)	0.92 (0.77–1.10)	0.92 (0.77–1.10)	0.91 (0.76–1.09)	0.89 (0.74–1.07)	0.93 (0.78–1.12)	0.90 (0.75–1.08)
Iran	906	22.7	67.3	1.66 (1.42–1.95)	1.56 (1.33–1.83)	1.73 (1.48–2.04)	1.65 (1.40–1.93)	1.75 (1.49–2.05)	1.71 (1.46–2.01)	1.75 (1.49–2.06)	1.31 (1.11–1.54)
Afghanistan	860	20.0	76.4	1.29 (1.09–1.53)	1.42 (1.20–1.68)	1.35 (1.14–1.60)	1.27 (1.07–1.51)	1.36 (1.15–1.61)	1.32 (1.11–1.56)	1.42 (1.19–1.69)	0.91 (0.76–1.08)
Morocco	472	18.6	77.8	1.17 (0.93–1.48)	1.11 (0.88–1.41)	1.19 (0.94–1.50)	1.12 (0.90–1.42)	1.23 (0.97–1.55)	1.22 (0.96–1.54)	1.14 (0.88–1.46)	0.95 (0.74–1.21)

^a^Stated as a percentage of the total number of deliveries

^b^All models adjusted for year of birth

^c^Gestational hypertensive disorders

Adjustment for pre-pregnancy BMI slightly increased the estimates for women from Vietnam, Thailand, the Philippines and China. For women from China the estimates changed from a risk similar to Danish-born women to an increased risk of emergency CS, increasing the risk among women from China with 16%.

Adjustment for maternal height considerably attenuated the RRRs for maternal country of birth and emergency CS for all immigrant groups. After adjustment for maternal height women from Somalia (RRR: 1.50, 95% CI = 1.29–1.75), the Philippines (RRR: 1.25, 95% CI = 1.10–1.44) and Iran (RRR: 1.31, 95% CI = 1.11–1.54) were the only groups with an increased risk ratio of emergency CS vs. VD compared to Danish-born women.

Separate adjustments for each of the explanatory variables did not affect the risk ratios of planned CS vs. VD, indicating that differences in planned CS could not be explained by these variables (Table 5).

**Table 5:** Relative risk ratios (RRR) and 95% confidence intervals for planned caesarean section (CS) versus vaginal delivery among primiparous women by maternal country of birth: Denmark 2004–2015

Maternal country of birth	Total number of deliveries n	Planned CS (%)^a^	Vaginal delivery (%)^a^	Adjusted for year of birth^b^	Adjusted for maternal age^b^	Adjusted for gestational age^b^	Adjusted for diabetes^b^	Adjusted for GHD^bc^	Adjusted for birthweight^b^	Adjusted for BMI^b^	Adjusted for height^b^
Denmark	272,888	5.4	78.5	1.00 (Ref.)	1.00 (Ref.)	1.00 (Ref.)	1.00 (Ref.)	1.00 (Ref.)	1.00 (Ref.)	1.00 (Ref.)	1.00 (Ref.)
Ex-Yugoslavia	2699	5.1	79.1	0.93 (0.78–1.11)	1.02 (0.86–1.22)	0.92 (0.76–1.10)	0.93 (0.78–1.10)	0.93 (0.78–1.11)	0.93 (0.78–1.11)	0.95 (0.80–1.14)	0.89 (0.75–1.07)
Poland	2685	6.6	78.4	1.22 (1.05–1.43)	1.25 (1.07–1.46)	1.22 (1.05–1.43)	1.23 (1.05–1.43)	1.23 (1.05–1.43)	1.23 (1.05–1.44)	1.26 (1.07–1.47)	1.20 (1.02–1.40)
Turkey	1856	3.6	78.0	0.66 (0.52–0.85)	0.72 (0.56–0.92)	0.66 (0.51–0.84)	0.64 (0.50–0.82)	0.66 (0.52–0.85)	0.62 (0.48–0.80)	0.74 (0.58–0.95)	0.68 (0.53–0.87)
Iraq	1663	4.0	79.5	0.72 (0.57–0.93)	0.88 (0.68–1.13)	0.72 (0.56–0.92)	0.71 (0.55–0.91)	0.73 (0.57–0.93)	0.74 (0.58–0.94)	0.76 (0.59–0.98)	0.68 (0.53–0.87)
Germany	1500	5.5	79.5	1.00 (0.80–1.25)	0.88 (0.70–1.10)	1.00 (0.80–1.25)	1.00 (0.80–1.25)	1.00 (0.80–1.25)	1.01 (0.80–1.26)	1.02 (0.81–1.28)	1.00 (0.80–1.26)
Norway	1486	4.8	82.9	0.83 (0.66–1.06)	0.78 (0.61–0.99)	0.84 (0.66–1.07)	0.84 (0.66–1.07)	0.84 (0.66–1.06)	0.84 (0.66–1.07)	0.85 (0.67–1.09)	0.83 (0.65–1.06)
Sweden	1403	6.4	78.8	1.18 (0.95–1.46)	1.04 (0.84–1.29)	1.19 (0.96–1.47)	1.20 (0.97–1.49)	1.18 (0.95–1.46)	1.19 (0.96–1.48)	1.22 (0.98–1.52)	1.16 (0.93–1.44)
Romania	1346	6.3	77.9	1.18 (0.95–1.48)	1.20 (0.96–1.50)	1.17 (0.94–1.46)	1.18 (0.95–1.48)	1.19 (0.95–1.48)	1.20 (0.96–1.50)	1.25 (1.00–1.56)	1.15 (0.92–1.44)
China	1292	3.7	80.6	0.67 (0.50–0.90)	0.63 (0.47–0.84)	0.66 (0.50–0.89)	0.65 (0.48–0.86)	0.67 (0.51–0.90)	0.68 (0.51–0.91)	0.66 (0.49–0.90)	0.57 (0.42–0.77)
Philippines	1127	5.3	65.7	1.18 (0.91–1.53)	1.13 (0.87–1.48)	1.16 (0.89–1.51)	1.15 (0.88–1.49)	1.18 (0.91–1.54)	1.20 (0.92–1.56)	1.23 (0.94–1.61)	0.98 (0.75–1.29)
Thailand	1088	6.8	70.1	1.40 (1.11–1.78)	1.26 (0.99–1.60)	1.37 (1.08–1.74)	1.38 (1.09–1.76)	1.41 (1.11–1.79)	1.41 (1.11–1.79)	1.54 (1.21–1.97)	1.28 (1.00–1.63)
Pakistan	1070	3.8	79.2	0.70 (0.51–0.96)	0.76 (0.56–1.04)	0.69 (0.51–0.95)	0.67 (0.49–0.91)	0.70 (0.51–0.96)	0.71 (0.52–0.97)	0.68 (0.49–0.96)	0.62 (0.44–0.86)
Somalia	956	2.9	71.7	0.59 (0.41–0.86)	0.70 (0.48–1.02)	0.62 (0.43–0.91)	0.58 (0.40–0.85)	0.60 (0.40–0.86)	0.60 (0.41–0.87)	0.65 (0.44–0.95)	0.60 (0.41–0.87)
Vietnam	953	3.5	76.8	0.65 (0.46–0.92)	0.63 (0.44–0.89)	0.63 (0.45–0.90)	0.64 (0.45–0.91)	0.65 (0.46–0.93)	0.63 (0.44–0.90)	0.72 (0.51–1.02)	0.58 (0.41–0.82)
Lebanon	926	2.8	84.7	0.48 (0.32–0.71)	0.58 (0.39–0.86)	0.47 (0.32–0.70)	0.48 (0.32–0.71)	0.48 (0.33–0.71)	0.49 (0.33–0.73)	0.50 (0.34–0.75)	0.45 (0.30–0.67)
Iceland	910	2.5	82.2	0.44 (0.29–0.67)	0.47 (0.31–0.72)	0.44 (0.29–0.67)	0.45 (0.30–0.68)	0.44 (0.29–0.67)	0.44 (0.29–0.67)	0.44 (0.29–0.67)	0.43 (0.28–0.66)
Iran	906	9.9	67.3	2.14 (1.71–2.67)	1.94 (1.55–2.40)	2.11 (1.69–2.64)	2.11 (1.69–2.63)	2.15 (1.72–2.68)	2.17 (1.74–2.71)	2.18 (1.74–2.74)	1.99 (1.59–2.50)
Afghanistan	860	3.6	76.4	0.68 (0.48–0.98)	0.79 (0.55–1.14)	0.68 (0.47–0.97)	0.67 (0.47–0.96)	0.69 (0.48–0.99)	0.70 (0.49–1.01)	0.69 (0.47–1.01)	0.59 (0.40–0.86)
Morocco	472	3.6	77.8	0.67 (0.41–1.09)	0.61 (0.37–1.00)	0.69 (0.42–1.12)	0.63 (0.39–1.02)	0.68 (0.41–1.10)	0.69 (0.42–1.12)	0.70 (0.42–1.16)	0.65 (0.39–1.07)

^a^Stated as a percentage of the total number of deliveries

^b^All models adjusted for year of birth

^c^Gestational hypertensive disorders

## Discussion

### Main findings

Findings from this study reveal substantial variation in the risk of CS among women from the largest immigrant groups in Denmark compared to national born women in the period 2004–2015. Increased risk ratios for emergency CS vs. VD were found for women from Turkey, the Philippines, Thailand, Somalia, Vietnam, Iran and Afghanistan compared to Danish-born women, whereas the risk ratios for planned CS vs. VD were lower among the majority of immigrant groups, but increased among women born in Poland, Thailand and Iran.

None of the studied demographic, medical and obstetric factors affected the risk ratio of planned CS vs. VD. However, maternal height contributed with varying strength to the risk ratios for emergency CS vs. VD for all immigrant groups, but the increased risk ratios persisted among women from Somalia, the Philippines and Iran.

### Interpretation

Several international studies have identified differences in CS rates among non-Western immigrants and national born women in Western industrialized populations [6, 10]. However, few previous studies differentiate according to type of CS and use various definitions of ethnicity, making comparison difficult.

Consistent with our findings, previous studies differentiating according to type of CS and maternal country of origin, have reported an increased risk of emergency CS among immigrant women from Somalia and the Horn of Africa [11–16]. Also consistent with our findings, a Swedish study from 2017 reported an increased risk of both planned and emergency CS among women from Thailand and Iran [16].

In the present study, differences in length of gestation, maternal age at delivery, diabetes, gestational hypertensive disorders, fetal birth weight and pre-pregnancy BMI, did not seem to explain differences in either emergency or planned CS. Adjustments for maternal height, however, led to a substantial attenuation of the risk estimates for emergency CS, especially among women with East Asian origin. After adjustment for maternal height, the increased risk ratio of emergency CS vs. VD only persisted among women from Somalia (RRR: 1.50; 95% CI: 1.29–1.75), the Philippines (RRR 1.25; 95% CI: 1.10–1.44) and Iran (RRR 1.31; 95% CI: 1.11–1.54).

With an average height of 155–158 cm, East Asian women had the lowest heights, 10–13 cm below the average height of Danish-born women (Tables 1 and 2). Short stature has been associated with CS due to labor dystocia and cephalopelvic disproportion [17–19]. A Norwegian study found that among immigrant women from the Philippines fetopelvic disproportion was the indication for CS in 40% of planned CS and 60% of emergency CS cases [12]. In present study, 80% of women born in Thailand and the Philippines had partners of Danish origin. Both maternal and paternal height have genetic effects on the fetal size of offspring and a large average parental height difference is linked to increased risk of complicated births [20]. Thus, the increased risk ratio of emergency CS vs. VD among women from Thailand and the Philippines might be explained by cephalopelvic disproportion due to short stature and large for gestational age (LGA) birth weight due to paternal influences. Increased attention from clinicians about the possible need for induction of labor or a planned CS among short stature women might help prevent an emergency CS due to cephalopelvic disproportion. More research providing prediction models for the risk of CS taking factors such as maternal height, paternal origin and estimated fetal birthweight into account could be helpful in clinical decision-making.

Patient preference has been considered a potential explanation for differences in CS rates. A Norwegian study found that immigrant mothers’ preferred mode of delivery is influenced by the CS rate in their country of origin [21]. Iran has one of the highest CS rates in the world [2]; a fear of vaginal delivery and social norms are suggested reasons [22]. A study from Tehran found that high age at marriage and high level of education was associated with preference for delivery by CS [23]. Likewise, our results demonstrate a higher maternal age at first delivery and generally higher education level among immigrants from Iran. Thus, social and cultural factors are possible explanations for the observed elevated risk of planned and emergency CS among Iranian women. However, lack of information about CS on maternal request did not enable us to investigate this further.

With a proportion of 2.9% of all deliveries, Somali women had one of the lowest risks of planned CS and additionally one of the highest risks of emergency CS. This was unexplained by common risk factors for CS, indicating the influence of other factors. High rates of emergency CS, but low rates of planned CS may be a sign that this group is not being assigned to a medically indicated CS, resulting instead to an emergency CS.

This vulnerability of Somali-born women in relation to emergency CS is consistent with previous studies [11–16]. Qualitative studies have shown that this group of women perceive CS with significant fear [24–26]. Social, cultural and linguistic barriers have been reported to lead to mutual misunderstanding and mistrust between the pregnant and delivering women and the health care providers [5, 6, 27]. It has also been documented that these barriers in a Nordic context are related to delays in the initiation of optimal care, resulting in an increased risk of acute interventions and poor perinatal outcome [28, 29]. Thus, suboptimal care for migrant women, maybe due to cultural and linguistic barriers and inadequate cross-cultural communication skills among health care providers might play a role.

High prevalence of female genital cutting has been suggested as an explanation of the high emergency CS rates in this group [30, 31], this is, however an issue that could be addressed prenatally.

Studies have reported that there is inadequate provision by health care services of good-quality interpreter services for migrants [32, 33]. Adequate use of interpreters during prenatal care and labor might be an important factor in preventing an overuse of emergency CS in certain ethnic groups. Additional, more detailed, studies investigating indications for CS are likely to be more informative than simply comparing CS rates and might provide important insights into possible interventions for reducing the increased risk of stillbirth and infant death reported among the Somali group in Denmark [34].

### Strengths and limitations

A main strength of this study is the design as a population-based register study minimizing the risk of selection bias and enabling data on a large set of covariates [35].

Our results showing the high heterogeneity according to maternal country of birth, highlight the risk of concealing unique between group differences when using broader definitions of ethnicity (regional geographical origin or general immigrant status). None of the women had missing information on country of origin and Statistics Denmark consider the quality of these data high [36], indicating low risk of bias due to misclassification by exposure status. Descendants were included in the group of Danish-born women, since they are born in Denmark. A Danish register study of ethnic differences in the use of prenatal care and the relation to perinatal deaths, showed substantial differences in the distribution of socio-economic parameters between descendants of non-Western origin and women of Danish origin [37]. Low socio-economic status might act as a proxy for other risk factors for CS including diabetes and abnormal BMI. Hence, the inclusion of descendants in the group of Danish-born women might have led to an underestimation of the association between maternal country of birth and the risk of CS. If descendants were included in the exposed group according to their mothers’ country of origin, descendants accounted for a substantial proportion (25–55%) of all deliveries for the Turkish, Ex-Yugoslavian, Pakistani, Lebanese and Moroccan group. For all other groups of national origin, the descendants constituted a small percentage. In total, however, deliveries by descendants only accounted for 2% of the total number of deliveries by Danish-born women. Additionally, the frequency of emergency and planned CS by women of Danish origin was the same regardless of inclusion or exclusion of deliveries by descendants, indicating no risk of bias (Additional files 1, 2 and 3).

Information about deliveries by CS was obtained from the Danish Medical Birth registry. A validation process has shown high consistency between information in patient records and this register [38]. Despite explicit guidelines for the definition of CS type, inconsistency might appear in certain clinical situations [38]; this is, however, unlikely to be related to maternal country of birth and will in most circumstance have been non-differential.

Previous studies have primarily used CS as a dichotomous outcome. Due to large differences in indications for performing either an emergency or planned CS, important information in relation to preventive strategies might be lost when not differentiating according to the type of CS.

In order to gain more insights into how demographic, medical and obstetric variables affected the association we made separate adjustments for these to test their importance for the observed ethnic differences in the risk of CS, and multiple adjustments were avoided.

## Conclusion

In conclusion, we have provided evidence of substantial variation in the risk ratio of respectively emergency and planned CS vs. VD among the largest groups of immigrants in Denmark relative to Danish-born women.

A number of known risk factors for CS did not seem to explain the observed ethnic differences in CS. However, findings suggest that increased focus on induction of labor and planning of CS could be justified among immigrant women of short stature to prevent emergency CS and we encourage further investigation within this area.

Factors such as suboptimal care for migrant women, maybe due to cultural and linguistic barriers, inadequate cross-cultural communication skills among health care providers and inadequate use of interpreters might likewise play a role. Further investigation of these factors might be of importance for furthering our understanding of the pathways leading to ethnic differences in CS.

## Additional files

Additional file 1: **Table S2.** Relative risk ratios (RRR) and 95% confidence intervals for respectively emergency caesarean section (CS) and planned CS versus vaginal delivery among primiparous women by maternal country of birth (analysis where deliveries by descendants of immigrants are excluded): Denmark 2004–2015. (DOCX 15 kb)

Additional file 2: **Table S3.** Relative risk ratios (RRR) and 95% confidence interval for emergency caesarean section (CS) versus vaginal delivery among primiparous women by maternal country of birth (analysis where deliveries by descendants of immigrants are excluded): Denmark 2004–2015. (DOCX 17 kb)

Additional file 3: **Table S4.** Relative risk ratios (RRR) and 95% confidence interval for planned caesarean section (CS) versus vaginal delivery among primiparous women by maternal country of birth (analysis where deliveries by descendants of immigrants are excluded): Denmark 2004–2015. (DOCX 17 kb)

## Abbreviations

CS: Caesarean section
ICD-10: International Classification of Disease codes
LGA: Large for gestational age
RRR: Relative risk ratios
VD: Vaginal delivery
WHO: World Health Organization

## References

[1] OECD. Health care activities. Health at a Glance 2015 - OECD Indicators. Paris: OECD Publishing; 2015. p. 114–5.

[2] World Health Organization. Health service coverage. World Health Statistics 2015. Geneva: WHO press; 2015. p. 89–100.

[3] Danish Health Authority. Kejsersnit 1973–2005 - Nye tal fra Sundhedsstyrelsen [Internet]. Copenhagen; 2005 . Available from: https://docplayer.dk/16530803-Nye-tal-fra-sundhedsstyrelsen-2005-19.html. [Cited 13 May 2019].

[4] WHO - Department of Reproductive Health and Research. WHO statement on caesarean section rates. Geneva: World Health Organization; 2015.

[5] Merry L, Semenic S, Gyorkos TW, Fraser W, Small R, Gagnon AJ (2016). International migration as a determinant of emergency caesarean. Women Birth.

[6] Merry L, Vangen S, Small R (2016). Caesarean births among migrant women in high-income countries. Best Pract Res Clin Obstet Gynaecol.

[7] United Nations - Department of Economic and Social Affairs. International migration report 2015 - highlights. New York: United Nations; 2016.

[8] Sobotka T (2008). Overview chapter 7: the rising importance of migrants for childbearing in Europe. Demogr Res.

[9] Statistics Denmark. Indvandrere i Danmark 2016 [Internet]. Copenhagen; 2016. Available from: http://www.dst.dk/Site/Dst/Udgivelser/GetPubFile.aspx?id=20704&sid=indv2016. [Cited 13 May 2019].

[10] Merry L, Small R, Blondel B, Gagnon AJ (2013). International migration and caesarean birth: a systematic review and meta-analysis. BMC Pregnancy Childbirth.

[11] Vangen S, Stoltenberg C, Johansen REB, Sundby J, Stray-Pedersen B (2002). Perinatal complications among ethnic Somalis in Norway. Acta Obstet Gynecol Scand.

[12] Vangen S, Stoltenberg C, Skrondal A, Magnus P, Stray-Pedersen B (2000). Cesarean section among immigrants in Norway. Acta Obstet Gynecol Scand.

[13] Bakken KS, Skjeldal OH, Stray-Pedersen B (2015). Immigrants from conflict-zone countries: an observational comparison study of obstetric outcomes in a low-risk maternity ward in Norway. BMC Pregnancy Childbirth.

[14] Råssjö EB, Byrskog U, Samir R, Klingberg-Allvin M (2013). Somali women’s use of maternity health services and the outcome of their pregnancies: a descriptive study comparing Somali immigrants with native-born Swedish women. Sex Reprod Healthc.

[15] Belihu Fetene B., Small Rhonda, Davey Mary-Ann (2016). Variations in first-time caesarean birth between Eastern African immigrants and Australian-born women in public care: A population-based investigation in Victoria. Australian and New Zealand Journal of Obstetrics and Gynaecology.

[16] Juárez SP, Small R, Hjern A, Schytt E (2017). Caesarean birth is associated with both maternal and paternal origin in immigrants in Sweden: a population-based study. Paediatr Perinat Epidemiol.

[17] Sheiner E, Levy A, Katz M, Mazor M (2005). Short stature-an independent risk factor for cesarean delivery. Eur J Obstet Gynecol Reprod Biol.

[18] Toh-Adam R, Srisupundit K, Tongsong T (2012). Short stature as an independent risk factor for cephalopelvic disproportion in a country of relatively small-sized mothers. Arch Gynecol Obstet.

[19] McGuinness BJ, Trivedi AN (1999). Maternal height as a risk factor for caesarean section due to failure to progress in labour. Aust N Z J Obstet Gynaecol.

[20] Stulp G, Verhulst S, Pollet TV, Nettle D, Buunk AP (2011). Parental height differences predict the need for an emergency caesarean section. PLoS One.

[21] Grytten J, Skau I, Sørensen R (2013). Do mothers decide?: the impact of preferences in healthcare. J Hum Resour.

[22] Latifnejad-Roudsari R, Zakerihamidi M, Merghati-Khoei E, Kazemnejad A (2014). Cultural perceptions and preferences of Iranian women regarding cesarean delivery. Iran J Nurs Midwifery Res.

[23] Ghotbi F, Sene AA, Azargashb E, Shiva F, Mohtadi M, Zadehmodares S (2014). Women’s knowledge and attitude towards mode of delivery and frequency of cesarean section on mother’s request in six public and private hospitals in Tehran, Iran, 2012. J Obstet Gynaecol Res.

[24] Brown E, Carroll J, Fogarty C, Holt C (2010). “They get a C-section . . . They Gonna die”: Somali Women’s fears of obstetrical interventions in the United States. J Transcult Nurs.

[25] Essén B, Binder P, Johnsdotter S (2011). An anthropological analysis of the perspectives of Somali women in the west and their obstetric care providers on caesarean birth. J Psychosom Obstet Gynaecol.

[26] Essén B, Johnsdotter S, Hovelius B, Gudmundsson S, Sjöberg NO, Friedman J (2000). Qualitative study of pregnancy and childbirth experiences in Somalian women resident in Sweden. BJOG An Int J Obstet Gynaecol.

[27] Binder P, Johnsdotter S, Essén B (2012). Conceptualising the prevention of adverse obstetric outcomes among immigrants using the “three delays” framework in a high-income context. Soc Sci Med.

[28] Essén B, Bödker B, Sjöberg NO, Langhoff-Roos J, Greisen G, Gudmundsson S (2002). Are some perinatal deaths in immigrant groups linked to suboptimal perinatal care services?. BJOG An Int J Obstet Gynaecol.

[29] Saastad E, Vangen S, Frøen JF (2007). Suboptimal care in stillbirths - a retrospective audit study. Acta Obstet Gynecol Scand.

[30] Wuest S, Raio L, Wyssmueller D, Mueller MD, Stadlmayr W, Surbek DV (2009). Effects of female genital mutilation on birth outcomes in Switzerland. BJOG An Int J Obstet Gynaecol.

[31] Banks E, Meirik O, Farley T, Akande O, Bathija H, Ali M (2006). Female genital mutilation and obstetric outcome: WHO collaborative prospective study in six African countries. Lancet.

[32] Small R, Roth C, Raval M, Shafiei T, Korfker D, Heaman M (2014). Immigrant and non-immigrant women’s experiences of maternity care: a systematic and comparative review of studies in five countries. BMC Pregnancy Childbirth.

[33] Binder P, Borné Y, Johnsdotter S, Essén B (2012). Shared language is essential: communication in a multiethnic obstetric care setting. J Health Commun.

[34] Villadsen SF, Mortensen LH, Andersen AMN (2009). Ethnic disparity in stillbirth and infant mortality in Denmark 1981-2003. J Epidemiol Community Health.

[35] Thygesen LC, Ersbøll AK (2014). When the entire population is the sample: strengths and limitations in register-based epidemiology. Eur J Epidemiol.

[36] Statistics Denmark (2016). Documentation of statistics for Immigrants and Descendants 2016 [Internet].

[37] Pedersen GS. Reproductive- and early child health among immigrants: PhD Thesis. Faculty of Health Science. University of Southern Denmark; 2013.

[38] Langhoff-Roos J, Rasmussen S. Validering af landspatientregistret (LPR) mhp. obstetrisk forskning og kvalitetssikring. Copenhagen: Sundhedsstyrelsen; 2003.

[39] National Comitee on Health Research Ethics (2018). Act on Research Ethics Review of Health Research Projects. Part 4, Section 14 (2) [Internet].

