# Supplementary material for: Ethnic differences in the risk of caesarean section: a Danish population-based register study 2004–2015
Source: BMC Pregnancy Childbirth. 2019 Jun 4;19:194. doi: 10.1186/s12884-019-2331-6 (PMC6549278; doi:10.1186/s12884-019-2331-6)
Supplement: Supplementary file 1 — Table S2. Relative risk ratios (RRR) and 95% confidence intervals for respectively emergency caesarean section (CS) and planned CS versus vaginal delivery among primiparous women by maternal country of birth (analysis where deliveries by descendants of immigrants are excluded): Denmark 2004–2015. (DOCX 15 kb) [file 12884_2019_2331_MOESM1_ESM.docx]

**Additional file 1**

| **Table S2. Relative risk ratios (RRR) and 95 % confidence intervals for respectively emergency caesarean section (CS) and planned CS versus vaginal delivery among primiparous women by maternal country of birth (excluding descendants): Denmark 2004-2015** | | | | | | |
| --- | --- | --- | --- | --- | --- | --- |
|  |  |  |  |  | **Emergency CS** | **Planned CS** |
| **Maternal country of birth** | **Total number of deliveries n** | **Emergency CS (%)**^a^ | **Planned**  **CS**  **(%)**^a^ | **Vaginal delivery (%)**^a^ | **Adjusted** **for**  **year of birth** | **Adjusted** **for**  **year of birth** |
| Denmark | 267,236 | 16.1 | 5.5 | 78.5 | 1.00 (Ref.) | 1.00 (Ref.) |
| Ex-Yugoslavia | 2,699 | 15.9 | 5.1 | 79.1 | 0.98 (0.88-1.09) | 0.92 (0.78-1.10) |
| Poland | 2,685 | 15.0 | 6.6 | 78.4 | 0.94 (0.85-1.05) | 1.21 (1.04-1.42) |
| Turkey | 1,856 | 18.4 | 3.6 | 78.0 | 1.15 (1.02-1.29) | 0.66 (0.51-0.84) |
| Iraq | 1,663 | 16.5 | 4.0 | 79.5 | 1.02 (0.89-1.16) | 0.72 (0.56-0.92) |
| Germany | 1,500 | 15.1 | 5.5 | 79.5 | 0.93 (0.81-1.07) | 0.99 (0.79-1.24) |
| Norway | 1,486 | 12.3 | 4.8 | 82.9 | 0.72 (0.62-0.85) | 0.83 (0.65-1.05) |
| Sweden | 1,403 | 14.8 | 6.4 | 78.8 | 0.92 (0.79-1.06) | 1.17 (0.94-1.45) |
| Romania | 1,346 | 15.8 | 6.3 | 77.9 | 1.01 (0.87-1.17) | 1.17 (0.94-1.46) |
| China | 1,292 | 15.7 | 3.7 | 80.6 | 0.96 (0.83-1.12) | 0.67 (0.50-0.89) |
| Philippines | 1,127 | 29.0 | 5.3 | 65.7 | 2.18 (1.91-2.48) | 1.17 (0.90-1.52) |
| Thailand | 1,088 | 23.1 | 6.8 | 70.1 | 1.61 (1.39-1.86) | 1.39 (1.10-1.77) |
| Pakistan | 1,070 | 17.0 | 3.8 | 79.2 | 1.05 (0.90-1.24) | 0.70 (0.51-0.96) |
| Somalia | 956 | 25.4 | 2.9 | 71.7 | 1.73 (1.50-2.01) | 0.59 (0.40-0.86) |
| Vietnam | 953 | 19.7 | 3.5 | 76.8 | 1.25 (1.07-1.47) | 0.65 (0.46-0.92) |
| Lebanon | 926 | 12.5 | 2.8 | 84.7 | 0.72 (0.59-0.87) | 0.48 (0.32-0.71) |
| Iceland | 910 | 15.3 | 2.5 | 82.2 | 0.90 (0.75-1.08) | 0.44 (0.29-0.67) |
| Iran | 906 | 22.7 | 9.9 | 67.3 | 1.66 (1.41-1.94) | 2.12 (1.71-2.65) |
| Afghanistan | 860 | 20.0 | 3.6 | 76.4 | 1.29 (1.09-1.52) | 0.68 (0.47-0.97) |
| Morocco | 472 | 18.6 | 3.6 | 77.8 | 1.17 (0.93-1.48) | 0.67 (0.41-1.09) |
| ^a^ Stated as a percentage of the total number of deliveries | | | | |  |  |
