# Supplementary material for: Ethnic differences in the risk of caesarean section: a Danish population-based register study 2004–2015
Source: BMC Pregnancy Childbirth. 2019 Jun 4;19:194. doi: 10.1186/s12884-019-2331-6 (PMC6549278; doi:10.1186/s12884-019-2331-6)
Supplement: Supplementary file 2 — Table S3. Relative risk ratios (RRR) and 95% confidence interval for emergency caesarean section (CS) versus vaginal delivery among primiparous women by maternal country of birth (analysis where deliveries by descendants of immigrants are excluded): Denmark 2004–2015. (DOCX 17 kb) [file 12884_2019_2331_MOESM2_ESM.docx]

**Additional file 2**

| **Table S3. Relative risk ratios (RRR) and 95 % confidence interval for emergency caesarean section (CS) versus vaginal delivery among primiparous women by maternal country of birth (excluding descendants): Denmark 2004-2015** | | | | | | | | | | | | |  |  |  |  |  |  |  |  |
| --- | --- | --- | --- | --- | --- | --- | --- | --- | --- | --- | --- | --- | --- | --- | --- | --- | --- | --- | --- | --- |
| **Maternal country of birth** | **Total number of deliveries n** | **Emergency CS**  **(%)**^a^ | | **Vaginal delivery (%)**^a^ | **Adjusted for year of birth**^b^ | | **Adjusted for maternal age**^b^ | | | **Adjusted for gestational age**^b^ | **Adjusted for diabetes**^b^ | **Adjusted for GHD**^b^**^c^** | | **Adjusted for birthweight**^b^ | | **Adjusted for BMI**^b^ | | **Adjusted for height**^b^ | |  |
| Denmark | 267,236 | 16.1 | | 78.5 | 1.00 (Ref.) | | 1.00 (Ref.) | | | 1.00 (Ref.) | 1.00 (Ref.) | 1.00 (Ref.) | | 1.00 (Ref.) | | 1.00 (Ref.) | | 1.00 (Ref.) | |  |
| Ex-Yugoslavia | 2,699 | 15.9 | | 79.1 | 0.98 (0.88-1.09) | | 1.05 (0.94-1.16) | | 0.99 (0.89-1.10) | | 0.98 (0.88-1.09) | 1.02 (0.92-1.14) | | | 1.02 (0.92-1.14) | | 1.06 (0.95-1.18) | | 0.89 (0.80-0.98) | |
| Poland | 2,685 | 15.0 | | 78.4 | 0.94 (0.85-1.05) | | 0.96 (0.86-1.07) | | 0.96 (0.86-1.07) | | 0.95 (0.85-1.05) | 0.97 (0.87-1.08) | | | 0.97 (0.87-1.08) | | 1.03 (0.93-1.15) | | 0.88 (0.79-0.98) | |
| Turkey | 1,856 | 18.4 | | 78.0 | 1.15 (1.02-1.29) | | 1.22 (1.08-1.37) | | 1.17 (1.04-1.32) | | 1.12 (1.00-1.27) | 1.19 (1.06-1.34) | | | 1.15 (1.02-1.29) | | 1.18 (1.04-1.34) | | 0.92 (0.81-1.04) | |
| Iraq | 1,663 | 16.5 | | 79.5 | 1.02 (0.89-1.16) | | 1.16 (1.01-1.32) | | 1.05 (0.92-1.19) | | 1.00 (0.88-1.14) | 1.07 (0.94-1.22) | | | 1.02 (0.90-1.17) | | 1.06 (0.93-1.21) | | 0.76 (0.67-0.87) | |
| Germany | 1,500 | 15.1 | | 79.5 | 0.93 (0.81-1.07) | | 0.86 (0.75-1.00) | | 0.95 (0.82-1.09) | | 0.93 (0.81-1.08) | 0.96 (0.83-1.10) | | | 0.95 (0.82-1.09) | | 0.97 (0.84-1.12) | | 0.94 (0.81-1.08) | |
| Norway | 1,486 | 12.3 | | 82.9 | 0.72 (0.62-0.85) | | 0.70 (0.59-0.81) | | 0.72 (0.61-0.84) | | 0.73 (0.62-0.85) | 0.74 (0.63-0.87) | | | 0.74 (0.63-0.86) | | 0.78 (0.67-0.92) | | 0.73 (0.63-0.86) | |
| Sweden | 1,403 | 14.8 | | 78.8 | 0.92 (0.79-1.06) | | 0.85 (0.73-0.99) | | 0.92 (0.79-1.07) | | 0.93 (0.80-1.08) | 0.92 (0.79-1.07) | | | 0.90 (0.77-1.05) | | 0.99 (0.85-1.16) | | 0.89 (0.77-1.04) | |
| Romania | 1,346 | 15.8 | | 77.9 | 1.01 (0.87-1.17) | | 1.02 (0.88-1.18) | | 1.04 (0.90-1.21) | | 1.01 (0.90-1.17) | 1.06 (0.92-1.23) | | | 1.02 (0.88-1.19) | | 1.11 (0.96-1.29) | | 0.85 (0.73-0.99) | |
| China | 1,292 | 15.7 | | 80.6 | 0.96 (0.83-1.12) | | 0.92 (0.79-1.07) | | 1.00 (0.86-1.16) | | 0.94 (0.81-1.09) | 1.03 (0.88-1.20) | | | 0.98 (0.84-1.15) | | 1.15 (0.99-1.34) | | 0.73 (0.62-0.85) | |
| Philippines | 1,127 | 29.0 | | 65.7 | 2.18 (1.91-2.48) | | 2.13 (1.87-2.42) | | 2.22 (1.94-2.53) | | 2.14 (1.88-2.44) | 2.26 (1.98-2.58) | | | 2.23 (1.95-2.55) | | 2.52 (2.21-2.89) | | 1.24 (1.08-1.42) | |
| Thailand | 1,088 | 23.1 | | 70.1 | 1.61 (1.39-1.86) | | 1.51 (1.31-1.75) | | 1.65 (1.43-1.91) | | 1.59 (1.38-1.84) | 1.69 (1.47-1.96) | | | 1.63 (1.41-1.88) | | 1.90 (1.64-2.21) | | 1.04 (0.90-1.22) | |
| Pakistan | 1,070 | 17.0 | | 79.2 | 1.05 (0.90-1.24) | | 1.11 (0.94-1.30) | | 1.07 (0.91-1.26) | | 1.02 (0.87-1.20) | 1.08 (0.92-1.26) | | | 1.01 (0.85-1.19) | | 1.08 (0.91-1.28) | | 0.72 (0.61-0.86) | |
| Somalia | 956 | 25.4 | | 71.7 | 1.73 (1.50-2.01) | | 1.94 (1.67-2.25) | | 1.64 (1.41-1.91) | | 1.72 (1.48-1.99) | 1.70 (1.46-1.97) | | | 1.71 (1.47-1.99) | | 1.90 (1.63-2.21) | | 1.49 (1.28-1.74) | |
| Vietnam | 953 | 19.7 | | 76.8 | 1.25 (1.07-1.47) | | 1.22 (1.04-1.44) | | 1.29 (1.10-1.52) | | 1.24 (1.06-1.46) | 1.33 (1.13-1.56) | | | 1.24 (1.05-1.46) | | 1.52 (1.29-1.79) | | 0.77 (0.65-0.90) | |
| Lebanon | 926 | 12.5 | | 84.7 | 0.72 (0.59-0.87) | | 0.82 (0.67-0.99) | | 0.74 (0.61-0.90) | | 0.72 (0.59-0.87) | 0.76 (0.62-0.92) | | | 0.74 (0.61-0.90) | | 0.77 (0.63-0.94) | | 0.55 (0.45-0.68) | |
| Iceland | 910 | 15.3 | | 82.2 | 0.90 (0.75-1.08) | | 0.94 (0.78-1.13) | | 0.92 (0.76-1.10) | | 0.91 (0.76-1.10) | 0.91 (0.75-1.09) | | | 0.88 (0.73-1.06) | | 0.93 (0.77-1.12) | | 0.90 (0.75-1.08) | |
| Iran | 906 | 22.7 | | 67.3 | 1.66 (1.41-1.94) | | 1.56 (1.33-1.83) | | 1.73 (1.48-2.03) | | 1.64 (1.40-1.92) | 1.74 (1.49-2.04) | | | 1.71 (1.45-2.00) | | 1.75 (1.49-2.06) | | 1.29 (1.10-1.52) | |
| Afghanistan | 860 | 20.0 | | 76.4 | 1.29 (1.09-1.52) | | 1.42 (1.20-1.68) | | 1.35 (1.14-1.60) | | 1.27 (1.07-1.50) | 1.36 (1.15-1.61) | | | 1.31 (1.10-1.56) | | 1.41 (1.19-1.69) | | 0.89 (0.75-1.07) | |
| Morocco | 472 | 18.6 | | 77.8 | 1.17 (0.93-1.48) | | 1.11 (0.88-1.41) | | 1.18 (0.94-1.50) | | 1.12 (0.89-1.42) | 1.23 (0.97-1.55) | | | 1.21 (0.96-1.54) | | 1.13 (0.88-1.46) | | 0.94 (0.74-1.20) | |
| ^a^ Stated as a percentage of the total number of deliveries | | | | | | | |  |  |  |  |  |  |  |  |  |  |  |  |  |
| ^b^ All models adjusted for year of birth | | |  | | |  | |  |  |  |  |  |  |  |  |  |  |  |  |  |
| ^c^ Gestational hypertensive disorders | | |  | | |  | |  |  |  |  |  |  |  |  |  |  |  |  |  |
