# Supplementary material for: Ethnic differences in the risk of caesarean section: a Danish population-based register study 2004–2015
Source: BMC Pregnancy Childbirth. 2019 Jun 4;19:194. doi: 10.1186/s12884-019-2331-6 (PMC6549278; doi:10.1186/s12884-019-2331-6)
Supplement: Supplementary file 3 — Table S4. Relative risk ratios (RRR) and 95% confidence interval for planned caesarean section (CS) versus vaginal delivery among primiparous women by maternal country of birth (analysis where deliveries by descendants of immigrants are excluded): Denmark 2004–2015. (DOCX 17 kb) [file 12884_2019_2331_MOESM3_ESM.docx]

| **Additional file 3**  **Table S4. Relative risk ratios (RRR) and 95 % confidence intervals for planned caesarean section (CS) versus vaginal delivery among**  **primiparous women by maternal country of birth (excluding descendants): Denmark 2004-2015** | | | | | | | | | | | | | | | |  |  |  |  |
| --- | --- | --- | --- | --- | --- | --- | --- | --- | --- | --- | --- | --- | --- | --- | --- | --- | --- | --- | --- |
| **Maternal country of birth** | **Total number of deliveries n** | **Planned CS (%)**^a^ | | **Vaginal delivery (%)**^a^ | **Adjusted for year of birth**^b^ | | | **Adjusted for maternal age**^b^ | **Adjusted for gestational age**^b^ | **Adjusted for diabetes**^b^ | **Adjusted for GHD**^b^**^c^** | **Adjusted for birthweight**^b^ | | **Adjusted for BMI**^b^ | | | **Adjusted for height**^b^ | |  |
| Denmark | 267,236 | 5.5 | | 78.5 | 1.00 (Ref.) | | | 1.00 (Ref.) | 1.00 (Ref.) | 1.00 (Ref.) | 1.00 (Ref.) | | 1.00 (Ref.) | | 1.00 (Ref.) | | | 1.00 (Ref.) | |
| Ex-Yugoslavia | 2,699 | 5.1 | | 79.1 | 0.92 (0.78-1.10) | | | 1.02 (0.85-1.21) | 0.92 (0.77-1.09) | 0.92 (0.77-1.10) | 0.93 (0.78-1.10) | | 0.92 (0.77-1.10) | | 0.95 (0.79-1.13) | | | 0.88 (0.74-1.06) | |
| Poland | 2,685 | 6.6 | | 78.4 | 1.21 (1.04-1.42) | | | 1.25 (1.07-1.46) | 1.21 (1.04-1.41) | 1.22 (1.04-1.42) | 1.22 (1.04-1.42) | | 1.22 (1.04-1.43) | | 1.25 (1.07-1.46) | | | 1.19 (1.01-1.39) | |
| Turkey | 1,856 | 3.6 | | 78.0 | 0.66 (0.51-0.84) | | | 0.72 (0.56-0.92) | 0.65 (0.51-0.84) | 0.63 (0.49-0.81) | 0.66 (0.51-0.84) | | 0.62 (0.48-0.80) | | 0.74 (0.58-0.95) | | | 0.67 (0.52-0.87) | |
| Iraq | 1,663 | 4.0 | | 79.5 | 0.72 (0.56-0.92) | | | 0.87 (0.68-1.12) | 0.71 (0.55-0.91) | 0.70 (0.55-0.90) | 0.73 (0.56-0.93) | | 0.73 (0.57-0.94) | | 0.76 (0.59-0.97) | | | 0.67 (0.52-0.86) | |
| Germany | 1,500 | 5.5 | | 79.5 | 0.99 (0.79-1.24) | | | 0.87 (0.70-1.10) | 0.99 (0.79-1.24) | 0.99 (0.79-1.25) | 0.99 (0.79-1.24) | | 1.00 (0.80-1.25) | | 1.01 (0.81-1.27) | | | 0.99 (0.79-1.25) | |
| Norway | 1,486 | 4.8 | | 82.9 | 0.83 (0.65-1.05) | | | 0.78 (0.61-0.99) | 0.83 (0.66-1.06) | 0.84 (0.66-1.06) | 0.83 (0.65-1.06) | | 0.84 (0.66-1.07) | | 0.85 (0.67-1.08) | | | 0.82 (0.65-1.05) | |
| Sweden | 1,403 | 6.4 | | 78.8 | 1.17 (0.94-1.45) | | | 1.04 (0.84-1.29) | 1.18 (0.95-1.46) | 1.19 (0.96-1.48) | 1.17 (0.94-1.45) | | 1.18 (0.95-1.47) | | 1.21 (0.98-1.51) | | | 1.15 (0.92-1.43) | |
| Romania | 1,346 | 6.3 | | 77.9 | 1.17 (0.94-1.46) | | | 1.20 (0.96-1.50) | 1.16 (0.93-1.45) | 1.17 (0.94-1.46) | 1.18 (0.94-1.47) | | 1.19 (0.95-1.49) | | 1.24 (0.99-1.55) | | | 1.14 (0.91-1.42) | |
| China | 1,292 | 3.7 | | 80.6 | 0.67 (0.50-0.89) | | | 0.63 (0.47-0.84) | 0.66 (0.49-0.88) | 0.64 (0.48-0.86) | 0.67 (0.50-0.90) | | 0.67 (0.50-0.90) | | 0.66 (0.48-0.89) | | | 0.56 (0.41-0.76) | |
| Philippines | 1,127 | 5.3 | | 65.7 | 1.17 (0.90-1.52) | | | 1.13 (0.87-1.47) | 1.15 (0.89-1.50) | 1.14 (0.87-1.48) | 1.17 (0.90-1.53) | | 1.19 (0.91-1.55) | | 1.22 (0.93-1.60) | | | 0.97 (0.74-1.27) | |
| Thailand | 1,088 | 6.8 | | 70.1 | 1.39 (1.10-1.77) | | | 1.26 (0.99-1.60) | 1.36 (1.07-1.73) | 1.37 (1.08-1.75) | 1.40 (1.10-1.78) | | 1.40 (1.10-1.78) | | 1.53 (1.20-1.95) | | | 1.26 (0.99-1.61) | |
| Pakistan | 1,070 | 3.8 | | 79.2 | 0.70 (0.51-0.96) | | | 0.76 (0.55-1.04) | 0.69 (0.50-0.94) | 0.66 (0.48-0.91) | 0.70 (0.51-0.96) | | 0.70 (0.51-0.96) | | 0.68 (0.48-0.95) | | | 0.61 (0.43-0.85) | |
| Somalia | 956 | 2.9 | | 71.7 | 0.59 (0.40-0.86) | | | 0.70 (0.48-1.02) | 0.62 (0.42-0.90) | 0.58 (0.40-0.85) | 0.59 (0.40-0.86) | | 0.59 (0.41-0.87) | | 0.65 (0.44-0.94) | | | 0.59 (0.41-0.86) | |
| Vietnam | 953 | 3.5 | | 76.8 | 0.65 (0.46-0.92) | | | 0.63 (0.44-0.89) | 0.63 (0.44-0.89) | 0.64 (0.45-0.90) | 0.65 (0.46-0.92) | | 0.63 (0.44-0.90) | | 0.71 (0.50-1.01) | | | 0.57 (0.40-0.81) | |
| Lebanon | 926 | 2.8 | | 84.7 | 0.48 (0.32-0.71) | | | 0.58 (0.39-0.85) | 0.47 (0.32-0.70) | 0.48 (0.32-0.70) | 0.48 (0.32-0.71) | | 0.49 (0.33-0.73) | | 0.50 (0.33-0.74) | | | 0.45 (0.30-0.66) | |
| Iceland | 910 | 2.5 | | 82.2 | 0.44 (0.29-0.67) | | | 0.47 (0.31-0.71) | 0.44 (0.29-0.67) | 0.45 (0.30-0.68) | 0.44 (0.29-0.67) | | 0.44 (0.29-0.67) | | 0.44 (0.29-0.67) | | | 0.43 (0.28-0.66) | |
| Iran | 906 | 9.9 | | 67.3 | 2.12 (1.71-2.65) | | | 1.93 (1.54-2.42) | 2.09 (1.68-2.62) | 2.09 (1.68-2.61) | 2.13 (1.71-2.66) | | 2.16 (1.73-2.70) | | 2.17 (1.73-2.72) | | | 1.97 (1.57-2.47) | |
| Afghanistan | 860 | 3.6 | | 76.4 | 0.68 (0.47-0.97) | | | 0.79 (0.55-1.13) | 0.67 (0.47-0.96) | 0.66 (0.46-0.95) | 0.68 (0.48-0.98) | | 0.70 (0.49-1.00) | | 0.69 (0.47-1.01) | | | 0.58 (0.40-0.85) | |
| Morocco | 472 | 3.6 | | 77.8 | 0.67 (0.41-1.09) | | | 0.61 (0.37-0.99) | 0.68 (0.42-1.11) | 0.62 (0.38-1.01) | 0.67 (0.41-1.09) | | 0.68 (0.42-1.11) | | 0.70 (0.42-1.15) | | | 0.64 (0.39-1.06) | |
| ^a^ Stated as a percentage of the total number of deliveries | | | | | | | |  |  |  |  |  |  |  |  |  |  |  |  |
| ^b^ All models adjusted for year of birth | | | |  | | |  |  |  |  |  |  |  |  |  |  |  |  |  |
| ^c^ Gestational hypertensive disorders | | | |  | | |  |  |  |  |  |  |  |  |  |  |  |  |  |
